# A Homicide in Disguise: How the Autopsy Dug up Clues

**DOI:** 10.7759/cureus.24691

**Published:** 2022-05-03

**Authors:** Aiman Khurshid, Hafsa Ahmad, Asra A Jaffry, Maman Khurshid, Gulzar Ali

**Affiliations:** 1 Forensic Medicine, Civil Hospital Karachi, Karachi, PAK; 2 Internal Medicine, Dow University of Health Sciences, Civil Hospital Karachi, Karachi, PAK

**Keywords:** forensic autopsy, adult, human, asphyxia, drowning, hyoid bone

## Abstract

An autopsy is performed in the occurrence of an out-of-the-ordinary manner of death where the cause of death is unclear. Through a detailed medicolegal investigation, it differentiates homicide from suicides or accidents. However, some people do not acknowledge its importance due to the conflict between science and religion. This is especially true for countries with a lack of education and awareness. The family of the deceased may be unmindful of medicolegal matters and hesitate to allow for an autopsy. In the instance that burial takes place before an autopsy was performed, the medicolegal officer requests for an exhumation. It is the act of digging up a body from its grave to be examined in more detail. Such was the case in our study. A dead body was retrieved from a water channel in the Sindh province, assumed to have accidentally drowned. The family held the funeral before an autopsy was performed. Later, suspicions arose surrounding the death, so the body was exhumed. The soft tissues were decomposed and unidentifiable. The examination suggested strangulation owing to the pivotal discovery of a fractured hyoid bone at the tip of the greater horn of the right cornu. Chemical tests came out negative for intoxication. Therefore, the cause of death was concluded to be asphyxia due to throttling, secondary to hyoid bone fracture. Currently, technology was developed to introduce advanced tests in forensic sciences to differentiate multiple causes of drowning. However, the dissatisfactory budget limits forensic experts in their work. There is little use in testing for diatoms to rule out drowning, as it has been proved to show discrepancies sometimes leading to a false-positive result. Hence, alternative methods need to be explored for a more efficient approach to find the cause of death.

## Introduction

A medicolegal autopsy is performed in the case of an uncommon or unnatural death to rule out foul play. The first challenge is to present a valid reason for the need for an autopsy. Deaths in the rural areas of a low-income country must be assessed with caution. Forensic experts are met with resistance by family members due to the lack of education on the rights of the deceased. A cross-sectional study showed how a majority was under the impression that autopsy is synonymous with body mutilation and organ donation. This goes against their religious belief [1]. Furthermore, resources are scarce, and the budget is limited for advanced tests and analysis. Oftentimes, burial takes place before an autopsy can be conducted. In such a case, exhumation is performed. The body is dug out under supervision and placed on the table for forensic examination [2].

The next obstacle is differentiating death by accidental drowning from homicide. Drowning may be the result of an accident, suicide, or homicide. It may also just be a means to dispose of the body and conceal the cause of death. This causes difficulties for forensic experts in case of decomposition or dismemberment [3]. The autopsy findings may be broadly categorized into multiple causes, so each case should be considered in its individual context [4]. Hence, all suspicions involving the body and its surrounding should be thoroughly investigated. Relevant circumstances before and after the death may be noted and compared, explaining the event of the death. These include a detailed history of the deceased, auditory or visual evidence, the body’s site of retrieval, and the deceased’s mental state [5]. The pathologist is taken on board to search for even minor injuries or artifacts to rule out immersion. Therefore, a medicolegal autopsy is of paramount importance to decisively conclude the cause of death.

We present the case of a body retrieved from the water and buried without an autopsy. The cause of death was thought to be accidental drowning until the manner of death was found suspicious. So, the body was exhumed, and a detailed medicolegal investigation ensued.

## Case presentation

A day-old dead body of a 29-year-old male was retrieved from an irrigation channel in Thatta, Sindh. Initially, the deceased was found with blood dripping from his nose and mouth. The body was then quickly taken away by the deceased’s brother before a further examination could be performed. No sample was sent for chemical analysis. The cause of death was deemed an accidental drowning, and the case was closed. Burial took place on that same day without an autopsy conducted.

**Figure 1:**
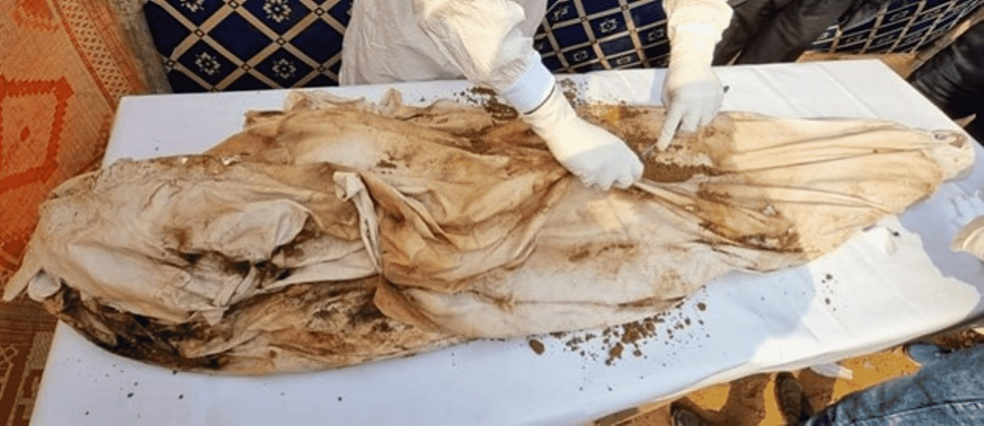
Cadaver wrapped in a dirty white shroud, with stains of mud and fluids of decomposition.

Five months and two weeks after the burial, rumors about the night of the death circulated around the family. They informed the police about their suspicions of a homicide. Hence, exhumation was requested, and the case was reopened by the investigating officer. The cadaver was dug out under supervision. On external examination, it was wrapped in a dirty white kafan (shroud to wrap a Muslim corpse for burial), stained with mud and decomposition fluids (Figure 1).

**Figure 2:**
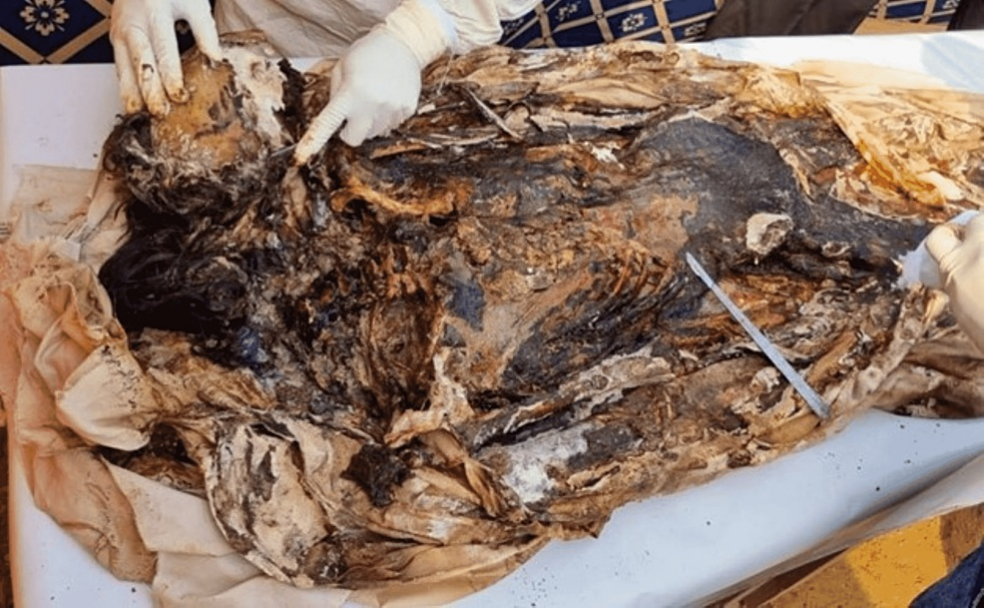
Cadaver found in an advanced stage of decomposition.

Unwrapped, the cadaver was found in an advanced stage of decomposition with unidentifiable features (Figure 2). The soft tissue had decomposed, and the teeth were loosely present in their sockets. The skeleton underneath was intact and subject to investigation. The skull showed congestion in both parietals, both temporals, and both mastoid regions. Blood clots were present in the sutures. However, no fracture was noted with all the facial bones intact.

**Figure 3:**
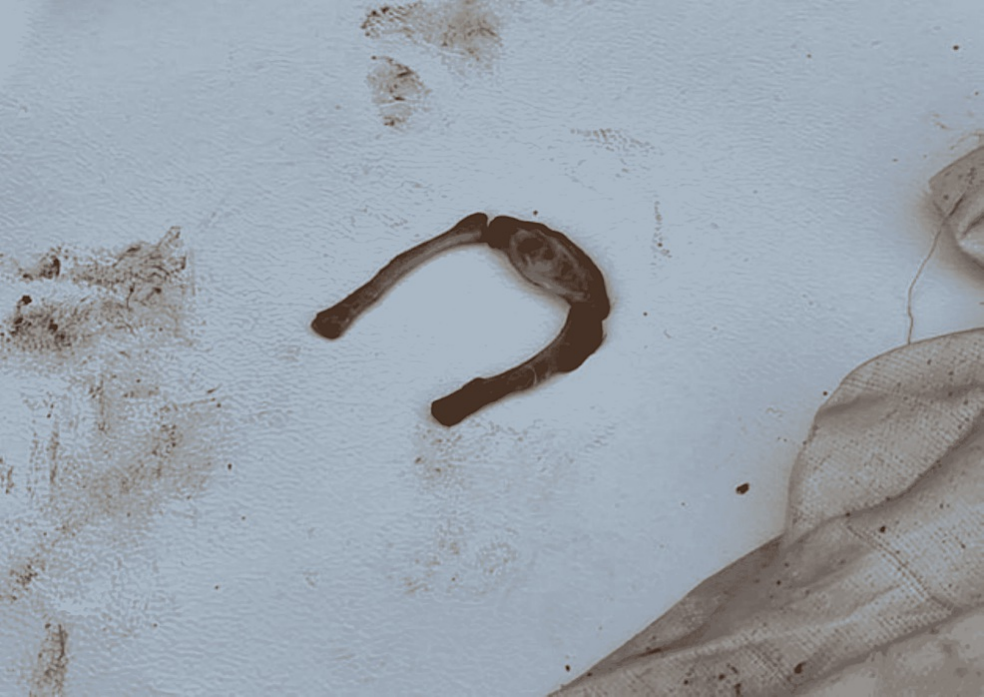
Hyoid bone fractured into three parts: right cornu, left cornu, and body.

A prominent point of examination was the hyoid bone, discovered in three pieces: the right and left cornu and the body (Figure 3). The tip of the right cornu, on the upper one-third of the greater horn, was fractured with congestion present.

Aside from this, all the cervical spines were intact. The remaining appendicular skeleton was unremarkable despite the decomposed soft tissues. On further examination, the chest and abdomen were also discovered in a state of decomposition. Small samples of mass from the two areas were preserved for chemical analysis. The fractured right cornu was also preserved as evidence of strangulation. The mud in the grave, a piece of kafan, and the right clavicle bone were also sent along with the visceral mass. No poison was detected, and intoxication was ruled out. Undoubtedly, the abovementioned examination described antemortem injuries.

Consequently, the exhumation board came to a unanimous conclusion that the cause of death was asphyxia due to throttling, secondary to a fracture in the tip of the right cornu of the hyoid bone.

## Discussion

In the above-reported case, exhumation was necessary to reach the correct cause of death. Despite the antemortem bleeding suggestive of homicide, accidental drowning had been considered initially, and the case was closed. This is common in areas with a low literacy rate [6]. Often, people are unaware that an autopsy makes it easier for the investigating officer to reach the culprit. They hesitate to consent for an autopsy unless the case is taken to court [6]. Similarly, the brother had taken the body away without an autopsy, maybe from fear of disobeying religion. The average person takes advice from religious officials. According to earlier studies, many showed a lack of understanding of the concept of autopsy. They believe autopsy causes an unfavorable delay in the burial [7]. However, most religious officials accept minimally invasive autopsy for the public’s benefit to find the cause of death in a homicide case [8]. The body was found in an advanced stage of decomposition. Fortunately, a study showed a negligible correlation between distorted postmortem findings affected by the decomposition fluids until some years have passed [9].

No marks on the skin of the neck were noted before the burial. Throttling suggests that the deceased may have shown resistance against the culprit. Had these been observed, redness and marks of fingers or nails on the neck of the deceased would have suggested throttling [10]. The fracture of the hyoid bone has been researched to be a large determinant in diagnosing manual strangulation, especially in the upper third of the greater horn [2]. Laryngeal and hyoid injuries are more common in cases of throttling, in the absence of ligature marks found mostly in suicidal hangings [11]. Furthermore, there was no DNA sample collected from the body initially. Any DNA samples collected could help differentiate between strangulation and drowning. Often, blood or skin epithelial cells may collect under the deceased’s nails during resistance so the culprit could be identified from DNA analysis.

A diatom test is a popular procedure to rule out drowning as it detects the presence of diatoms in the respiratory tract. However, it is also considered time-consuming and requires some developments in the method for more efficiency [12]. Hence, it was not conducted in our presented case that is from a low-income area with limited resources. Data may sometimes show discrepancies leading to a false-positive result due to antemortem diatom penetration into an organism or postmortem contamination. This could happen either during the submersion period or in diatom preparation [13].

Alternative methods could be performed ideally without budget limitations. According to research with a heart blood sample collected, unusually, high thyroglobulin concentration may be found indicating asphyxia involving neck compression [14]. Moreover, virtopsy may be performed with no bodily penetration and appease the public. Computed tomography (CT) displays clearer postmortem imaging and can differentiate if the victim was alive or dead at the time of drowning [15]. Polymerase chain reaction (PCR) is a tool in biopsy sampling to detect bacterioplankton in the organs of drowned bodies [16]. A diatom test was modified to show an increase in the reliability of results by implementing the microwave digestion-vacuum filtration-automated scanning electron microscopy (MD-VF-Auto SEM) in 128 cases [17]. Thus, Pakistan needs the concerned governing body to invest in the medicolegal department.

## Conclusions

Drowning may be a means to disguise homicide. The culprit may dispose of the body in the water and set the crime scene up as a suicide attempt or an accident. Therefore, medicolegal autopsies play a crucial role to rule out foul play in skeptical deaths to find the culprit. However, in third-world countries, owing to a widespread lack of education and awareness and staunch religious opinions, the local population hesitates to consent to an autopsy. Poor funding also contributes to the misdiagnosis. Important clues may go unnoticed, which could otherwise be found if a proper investigation is followed through. In such a case where the burial takes place before an autopsy could be conducted, exhumation is carried out. The body is dug out from the grave under the supervision of the magistrate and other investigating officers. It undergoes a thorough examination despite any decomposition or cremation.

In our case report, the hyoid bone was discovered in three pieces upon examination. More so, a fracture and congestion were present on the upper one-third of the greater horn of its right cornu. Despite no other findings, this fracture is fairly consistent in victims of manual strangulation. Furthermore, as discussed above, the diatom test may show unreliable results. Alternatively, the introduction of new methods, such as the aforementioned virtopsy by CT scan, PCR in biopsy sampling, and the modified MD-VF-Auto SEM, in forensic sciences will show a quicker and more efficient way to find the cause of death.
